# 4‐Methylumbelliferone Restores Age‐Related Changes in Perineuronal Nets, Memory, and Neuroinflammation

**DOI:** 10.1002/glia.70233

**Published:** 2026-09-30

**Authors:** Anda Cimpean, Jana Mizerakova, Noelia Martinez‐Varea, Dana Marekova, Lucia Machova Urdzikova, James W. Fawcett, Pavla Jendelova, Jessica C. F. Kwok

**Affiliations:** ^1^ Department of Neuroregeneration Institute of Experimental Medicine of the Czech Academy of Sciences Prague Czech Republic; ^2^ Department of Clinical Neurosciences, John Van Geest Centre for Brain Repair University of Cambridge Cambridge UK; ^3^ Faculty of Biological Sciences, University of Leeds Leeds UK

**Keywords:** 4‐methylumbelliferone, aging, cognitive decline, microglia, neuroinflammation, perineuronal nets, reactive astrocytes

## Abstract

Aging‐associated cognitive decline is closely linked to dysregulated glial activity and chronic neuroinflammation, which together constrain neuronal plasticity. Astrocytes and microglia are key regulators of the brain extracellular matrix (ECM), including perineuronal nets (PNNs), whose accumulation during aging contributes to reduced synaptic flexibility. Targeting ECM–glia interactions therefore represents a promising strategy to modulate neuroinflammatory and plasticity‐related processes. Here, we investigated the effects of long‐term oral administration of 4‐methylumbelliferone (4‐MU), an inhibitor of hyaluronan and chondroitin sulfate synthesis, on glial reactivity, ECM remodeling, and cognitive function in aged mice (20–22 months). Aging was associated with PNN remodeling alongside pronounced astrocytic and microglial activation and infiltration of peripheral immune cells. Chronic 4‐MU treatment reduced PNN accumulation to levels observed in younger (10‐month‐old) mice, indicating effective ECM remodeling. Notably, this was accompanied by a marked attenuation of astrocyte and microglial activation and a reduction in immune cell infiltration, suggesting a broad normalization of the neuroinflammatory milieu. Functionally, these glia‐associated changes were paralleled by improved recognition memory, with treated aged mice performing at or above the level of younger controls. Long‐term 4‐MU administration was well tolerated, with no evident adverse effects. Together, our findings identify ECM modulation as a mechanism to regulate glial reactivity and neuroimmune interactions in the aging brain. By targeting hyaluronan‐dependent pathways, 4‐MU links ECM remodeling to suppression of neuroinflammation and restoration of cognitive function, highlighting its therapeutic potential in age‐related neurodegenerative processes.

## Graphical abstract

Brain aging alters perineuronal nets and neuroinflammation. 4‐methylumbelliferone reversed these aging‐associated changes. 4‐methylumbelliferone reduced age‐associated cognitive decline. Six months of chronic oral treatment was well tolerated.

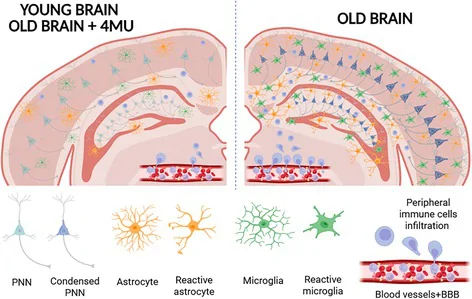

## 1 Introduction

Aging is characterized by a gradual decline in cognitive and motor functions, reducing the ability to learn, adapt to environmental changes, and recover from damage, impairments which are largely conserved across species. Aging is accompanied by a progressive loss of neuroplasticity within the central nervous system (CNS) (Brito et al. 2023). This reduction in plasticity reflects the convergence of multiple cellular, molecular, and structural processes. Among the key contributors are the accumulation of perineuronal nets (PNNs) (Karetko‐Sysa et al. 2014; Foscarin et al. 2017; Mafi et al. 2020; Lehner et al. 2024) and an increase in neuroinflammation (Patterson 2015; Tamatta et al. 2025).

PNNs are specialized extracellular matrix (ECM) structures that enwrap specific subgroups of neurons, most prominently parvalbumin‐positive (PV+) inhibitory interneurons (Lupori et al. 2023). They form unique mesh‐like pericellular coats that regulate the diffusion of neurotransmitters, ions, growth factors, and metabolites, as well as the distribution and clustering of synaptic receptors, thereby exerting a profound influence on synaptic transmission and neuronal function. PNNs are composed of a hyaluronan (HA) backbone anchored to the neuronal surface by hyaluronic acid synthase (HAS). Chondroitin sulfate proteoglycans (CSPGs) and tenascins attach to HA and are stabilized by link proteins (Crtl1/Hapln1, Bral2/Hapln4) (Testa et al. 2019; Auer et al. 2025). They are considered central pillars of neuroplasticity. Functionally, PNNs play a central role in closing critical periods of increased neuroplasticity in the CNS. Condensation of PNNs toward the end of neurodevelopment is highly linked to reduced synaptic flexibility and impaired learning in aged subjects. Experimental degradation or remodeling of PNNs in adulthood has been shown to enhance neuroplasticity, resulting in increased associative, spatial, social, and auditory memory (Fawcett et al. 2022).

Beyond its structural role in regulating synaptic plasticity, HA‐rich ECM is increasingly recognized as a dynamic signaling platform that coordinates also glial activation (Peters and Sherman 2020), inflammatory signaling (Kuipers, Rieck, et al. 2016), and blood–brain barrier (BBB) integrity (Al‐Ahmad et al. 2019).

Brain aging is accompanied by profound changes in glial cells. These include altered morphology, increased numbers, and a shift toward reactive phenotypes. Within the tetrapartite synapse, glial reactivity and structural alterations disrupt synaptic homeostasis (Latham et al. 2023). Such changes contribute to reduced neuroplasticity, neuronal degradation, and increased vulnerability to neurodegenerative processes (Jurcau et al. 2024).

Aging is also associated with compromised BBB integrity, which facilitates T‐cell infiltration into the CNS. Infiltrating immune cells interact bidirectionally with resident glia, creating a feedforward loop of inflammation: activated glia release pro‐inflammatory cytokines (e.g., TNF‐α, IL‐1β, IL‐6), chemokines (e.g., CCL2), reactive oxygen species (ROS, NO), and matrix metalloproteinases (MMP‐2, MMP‐9), which increase BBB permeability, further promoting T‐cell entry and amplifying glial activation (Latham et al. 2023; Nevalainen et al. 2022; Zhang et al. 2022). Together, these changes contribute to “inflammaging,” a state of chronic low‐grade inflammation that negatively affects synaptic function and neuroplasticity. Therapeutic strategies in this context aim to mitigate detrimental inflammatory cascades while preserving or enhancing protective glial functions.

The coumarin derivative 4‐methylumbelliferone (4‐MU) is a small molecule naturally occurring in plants of the *Umbelliferae* and *Asteraceae* families. Already approved in Europe and Asia for hepatoprotective, antispasmodic, and choleretic use (Nagy et al. 2015), 4‐MU is recognized for its modulation on HA metabolism. We have recently shown that 4‐MU is also able to reduce the synthesis of chondroitin sulfate (CS) (Milton et al. 2023). Mechanistically, 4‐MU depletes UDP‐GlcA required for HA and CS synthesis, downregulates hyaluronan synthase enzymes (HAS2) expression, and upregulates hyaluronidase enzymes (Hyal1) expression (Fedorova et al. 2025). Our previous studies have shown that 4‐MU administration enhances neuroplasticity through PNN downregulation improving memory (Dubisova et al. 2022) and promotes serotonergic sprouting after spinal cord injury in rodents (Štepánková et al. 2023). Furthermore, 4‐MU has well‐established immunomodulatory properties, with studies demonstrating reduced inflammation in preclinical models of Types 1 and 2 diabetes, autoimmune arthritis, as well as lung, renal, and liver inflammatory pathologies (Fedorova et al. 2025). While its role in modulating immune responses within the CNS is less characterized, evidence indicates that 4‐MU can attenuate glial scar formation after spinal cord injury (Štepánková et al. 2023), decrease immunoreactivity and disease progression in multiple sclerosis mice models (Kuipers, Rieck, et al. 2016) and exert anti‐inflammatory effects in rodent models of ischemic stroke (Mirshekari Jahangiri et al. 2024; Tamouk et al. 2025). These findings make 4‐MU, particularly, relevant in the context of neuroinflammaging. Moreover, numerous studies have demonstrated that 4‐MU is a well‐tolerated compound with minimal adverse effects (Dubisova et al. 2022; Yates et al. 2015; Štěpánková et al. 2023), including a study reporting lifelong 4‐MU treatment in mice without major adverse effects (Nagy et al. 2024), while its washout profile further indicates that any minor side effects are reversible and do not persist following treatment cessation (Dubisova et al. 2022; Štěpánková et al. 2023).

In this study, we investigated whether modulation of HA and CS synthesis by 4‐MU can simultaneously influence PNNs, neuroinflammation, and cognitive function during aging. We show that 4‐MU reduces age‐related PNN accumulation, normalizes or further decreases neuroinflammation, and improves memory performance, highlighting its potential to target ECM‐associated mechanisms underlying cognitive decline in aging.

## 2 Materials and Methods

### 2.1 Animals

We used three groups of C57BL/6JOlaHsd mice. The “10 m” group (*n* = 5; three male and two female) entered the study at 4 months of age and was fed chocolate‐flavored chow (Sniff GmbH, Soest, Germany) (placebo) for 6 months, reaching 10 months of age at the end of the experiment. The “22 m” group entered the study at 14–16 months of age and was divided into two subgroups: control (*n* = 15; eight male and seven female) and treated (*n* = 15; eight male and seven female). The control subgroup received chocolate‐flavored chow, while the treated subgroup received chocolate‐flavored chow supplemented with 5% (w/w) 4‐MU (DbPharma France) corresponding to a daily dose of 36.9 μmol/g for 6 months. At the end of the experiment, the group was 20–22 months old. All procedures were conducted in compliance with the European Communities Council Directive of 22 September 2010 (2010/63/EU), adhered to the ARRIVE guidelines, and were approved by the Ethics Committee of the Institute of Experimental Medicine CAS, Prague, Czechia. Animals had ad libitum access to food and water and were maintained on a 12 h light/dark cycle (lights off at 7:30 p.m.). Behavioral testing was performed during the light phase. At the end of the treatment period, animals underwent behavioral assessment of memory and motor functions, were weighed, and then sacrificed for blood and urine collection and brain isolation. Blood was collected by cardiac puncture into BD Microtainer tubes. Samples for hematological analysis were drawn into K_2_EDTA‐coated tubes (#365974), and the following parameters were measured: leukocytes, erythrocytes, platelets, reticulocytes, neutrophils, lymphocytes, monocytes, eosinophils, and basophils. For biochemical analyses, blood was collected in serum‐separating tubes (#365963) and urine samples were obtained via bladder puncture to determine glucose and bile acid levels. All measurements were performed by Synlab (Munich, Germany).

### 2.2 Behavioral Tests

To evaluate the effects of 4‐MU on aging, a series of behavioral tests were performed. Memory performance was assessed using the spontaneous object recognition (SOR) task, while motor function was evaluated using the rotarod and grip strength tests, which probe coordination, joint mobility, and muscle integrity.

#### 2.2.1 SOR Task

The SOR task was performed in a Y‐maze, in which one arm served as the start arm (4.5 cm long, 8 cm wide) and the other two arms (10 cm long, 8 cm wide) were used for object presentation. Randomly shaped junk objects (~10 × 5 × 5 cm) were used as stimuli. All mice were habituated to the maze in two daily sessions prior to testing. During the sample phase, mice were allowed to explore two identical objects for 5 min. After a delay of 6 or 24 h in the home cage, the choice phase was conducted. This phase was procedurally identical to the sample phase, except that one of the familiar objects was replaced with a novel object. Mice were again allowed to explore for 5 min. Behavioral performance was video‐recorded and analyzed offline by an experimenter blinded to treatment. Exploration time was defined as the duration during which the mouse oriented toward and contacted the object. Instances in which animals climbed or sat on objects were excluded. A discrimination score was calculated as (time exploring the novel object − time exploring the familiar object)/(total exploration time). Thus, a score of 1 indicated exclusive exploration of the novel object, while a score of 0 indicated equal exploration of both objects. Statistical analysis was performed using one‐way ANOVA followed by Tukey's multiple comparisons test.

#### 2.2.2 Rotarod and Grip Test

Motor coordination and balance were assessed in the 22 m old group using a rotarod apparatus equipped with automatic timers and fall sensors (ROTA‐ROD 47700, UGO BASILE S.R.L., Italy) with a 7 cm diameter drum. Prior to testing, mice were habituated to remain on the stationary drum for 60 s and then pretrained at a constant speed of 5 rotations per minute (RPM) for 120 s. During the test phase, animals were tested at 10 RPM for up to 300 s in three consecutive daily sessions following training. The latency to fall was automatically recorded.

Muscle strength was assessed using a grip strength meter (BIO‐GS3, Bioseb, Vitrolles, France). Mice were tested for hind limb grip strength in three consecutive daily sessions. Each mouse was allowed to grasp the metal grid with its hind paws and was gently pulled backward until the grip was released; the maximal force exerted was recorded automatically. Statistical analysis was performed using an unpaired *t*‐test.

### 2.3 Tissue Preparation and Immunohistochemistry

Mice were deeply anesthetized with an intraperitoneal injection of an overdose of pentobarbital (Sigma‐Aldrich, St. Louis, MO, USA) and intracardially perfused with phosphate‐buffered saline (PBS), followed by 4% paraformaldehyde (PFA) in 0.1 M phosphate buffer. Heads were postfixed in 4% PFA for 1 week. Brains were then dissected from the skull and cryoprotected in increasing concentrations of sucrose (10%–30%, w/v). After cryoprotection, brains were embedded in O.C.T. compound (00411243, VWR), frozen, and coronally sectioned at 20 μm thickness using a cryostat (CryoStar NX70, Thermo Fisher Scientific, MA, USA). Sections were collected free‐floating in PBS and stored at −20°C in cryoprotectant solution until use. For immunohistochemistry, free‐floating sections were permeabilized with 0.5% (v/v) Triton X‐100 in PBS for 20 min at room temperature. Lipofuscin autofluorescence was quenched using 50 mM ammonium acetate buffer with CuSO₄. Endogenous biotin was blocked using an endogenous biotin blocking kit (ab64212, Abcam, Cambridge, UK). PNNs were visualized using *Wisteria floribunda* agglutinin (WFA), which binds *N*‐acetylgalactosamine β1 residues of glycoproteins within the neuronal ECM (1:150; biotinylated WFA, L1516, Sigma‐Aldrich). Nonspecific binding of WFA was blocked with 10% Chemiblocker (#2170, Merck, Darmstadt, Germany) in PBS containing 0.2% Triton X‐100 for 2 h at room temperature. Sections were incubated overnight at 4°C with primary antibodies against GFAP (chicken, 1:800, ab4674, Abcam), Iba1 (goat, 1:800, 019‐19741, Fujifilm), CD45 (rabbit, 1:300, bs‐10599R, Bioss), and biotinylated WFA. Nuclei were counterstained with DAPI (1:1000, D1306, Invitrogen). The following secondary antibodies were used: streptavidin–Alexa Fluor 488 (1:200, S32354, Life Technologies), donkey anti‐rabbit Alexa Fluor 594 (1:400, A‐31556, Thermo Fisher Scientific), goat anti‐chicken Alexa Fluor 564 (1:400, A‐11041, Thermo Fisher Scientific), and donkey anti‐goat Alexa Fluor 488 (1:400, A11055, Invitrogen). After staining, sections were mounted on glass slides, coverslipped with antifade mounting medium, and stored at 4°C until imaging.

### 2.4 Imaging and Analysis

Images were acquired using an Olympus SpinSR10 inverted fluorescence confocal spinning disk microscope. A ×20 objective was used for general imaging, and a ×40 objective was used for glia‐morphology analysis. WFA‐labeled PNNs were imaged in the somatosensory cortex and hippocampus, while inflammation‐related analyses were focused on the somatosensory cortex, corpus callosum, and hippocampus. Image analysis was performed using ImageJ (NIH) software. Staining was performed in parallel across all conditions. All experimental groups (10 m control, 22 m control, and 22 m treated) were processed together within the same staining batch using identical antibody solutions, incubation times, washing steps, and handling procedures. Imaging parameters were kept constant throughout acquisition within each staining batch, including laser power, detector gain, exposure time, pinhole, objective, and z‐step.

For PNN characterization, we manually quantified the number of neurons ensheathed by PNNs (PNN density). We also measured mean WFA fluorescence intensity and analyzed the intensity distribution by focusing on the top quartile (the 25% brightest pixels), thereby highlighting the most intensely labeled PNN structures. PNN thickness was estimated from fluorescence intensity profiles crossing the neuronal soma and calculated as the full width at half maximum (FWHM) of the profile.

Astrocytes were analyzed by GFAP immunostaining. The total number of GFAP‐positive cells and the mean GFAP signal intensity were quantified. Astrocytic morphology in the somatosensory cortex and hippocampus was assessed using Sholl analysis, in which concentric circles of increasing radius are drawn around the cell soma and the number of intersections between processes and circles is counted (starting radius: 3 μm; step size: 3 μm). This method provides a measure of branching complexity and arborization. In the corpus callosum, this analysis could not be performed due to the elongated morphology of astrocytes in this region. Instead, we quantified the cell body area, as soma hypertrophy is a well‐established feature of reactive astrocytes. Microglia were examined by Iba1 immunostaining. Similar to astrocytes, we quantified the total number of Iba1‐positive cells, the mean Iba1 fluorescence intensity, and microglial morphology using Sholl analysis (starting radius: 5 μm; step size: 3 μm). To evaluate peripheral immune cell infiltration, a combination of Iba1 and CD45 staining was used. Since CD45 labels both microglia and peripheral immune cells, cells positive for CD45 but negative for Iba1 were manually counted by a blinded experimenter. Statistical analyses were performed using ordinary one‐way ANOVA followed by Tukey's multiple comparisons test.

## 3 Results

### 3.1 4‐MU Treatment Counteracts Age‐Related Increase in PNNs and Enhances Object Recognition Memory in Aged Mice

In our previous work, we showed that oral 4‐MU treatment in 10‐month‐old adult mice reduced total CNS glycosaminoglycan levels, decreased PNN formation, modulated the expression of HA‐ and PNN‐related genes, and improved object recognition memory (Dubisova et al. 2022). In the present study, we sought to assess how PNNs change with aging and to evaluate the impact of long‐term 4‐MU treatment on these age‐related alterations.

We performed WFA staining for PNNs on coronal brain sections from 10‐month‐old adult mice (10 m), 22‐month‐old aged mice (22 m), and 22 m aged mice treated with 5% 4‐MU (w/w chow) for 6 months (22 m + 4MU). Our analysis focused on the somatosensory cortex and hippocampus, regions critical for cognitive function (Figure 1A).

**FIGURE 1:**
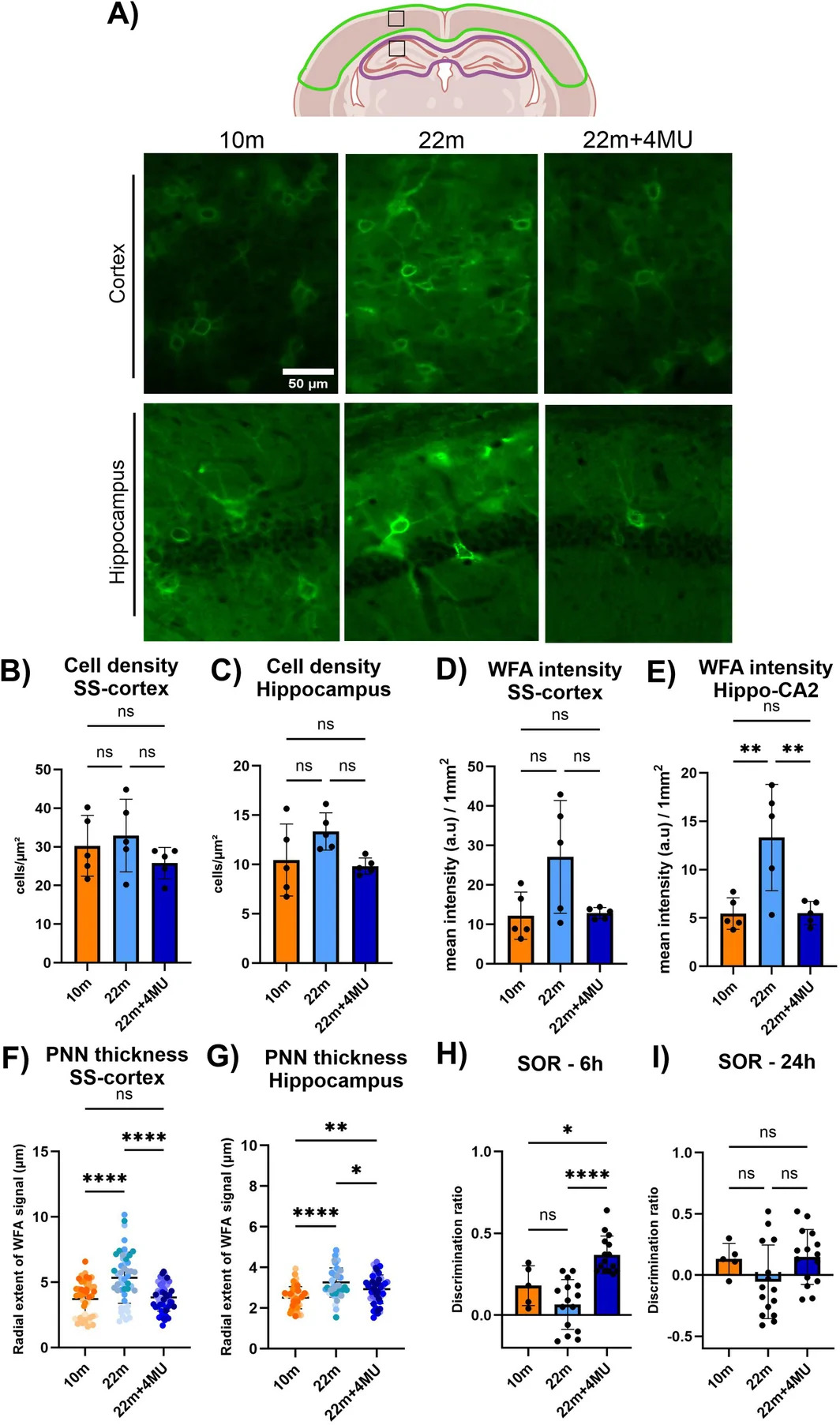
4‐MU attenuates age‐related PNN accumulation and improves memory performance. (A) Schematic representation of the brain regions analyzed, and images of WFA immunofluorescence in the cerebral cortex and hippocampus of 10‐month‐old (10 m), 22‐month‐old (22 m), and 22‐month‐old mice treated with 4‐MU for 6 months (22 m + 4MU). (B, C) Quantification of the number of neurons surrounded by PNNs in the cortex and hippocampus, respectively. (D, E) Mean PNN fluorescence intensity in cortex and hippocampus, respectively. (F, G) PNN thickness in cortex and hippocampus, respectively. Each color represents an individual cell (10 cells per animal; 50 cells per group). (H, I) SOR performance at 6 and 24 h retention intervals, respectively. Data are presented as mean ± SD and analyzed using ordinary one‐way ANOVA; *p < 0.05, **p < 0.01, ****p < 0.0001.

Regarding the proportion of neurons surrounded by PNNs, no major differences were observed between groups in the somatosensory cortex (10 m: 30 ± 7; 22 m: 32 ± 9; 22 m + 4MU: 25 ± 4) or hippocampus (10 m: 10 ± 3; 22 m: 13 ± 2; 22 m + 4MU: 9 ± 1) (Figure 1B,C).

In contrast, PNN mean intensity showed an increase in 22‐month‐old animals in the somatosensory cortex (10 m: 12.2; 22 m: 27.11; 22 m + 4MU: 12.8) and a significant increase in the CA2 region of the hippocampus (10 m: 5.4 vs. 22 m: 12.32, *p* = 0.0082), while 4‐MU treatment reduced intensity to levels comparable to those observed in 10‐month‐old animals (22 m + 4MU: 5.5, *p* = 0.0086) (Figure 1D,E).

To further characterize changes in WFA labeling, we analyzed the distribution of pixel intensities (Figure S1A). Aging was associated with a shift in WFA signal distribution from low‐intensity pixels toward intermediate intensity ranges, consistent with an overall increase in PNN‐associated staining intensity across the cortex. This shift was partially mitigated by 4‐MU treatment. Additionally, analysis of the highest intensity bin (Q4; brightest WFA signal) revealed an increase in both somatosensory cortex and hippocampus of 22‐month‐old animals (cortex: 10 m, 0.04 vs. 22 m, 0.11, *p* = 0.007; hippocampus: 10 m, 0.14 vs. 22 m, 0.53, *p* = 0.0058), which was reduced following 4‐MU treatment (22 m + 4MU cortex: 0.02, *p* = 0.001; hippocampus: 0.20, *p* = 0.0177), returning toward young levels (Figure S1B).

Finally, PNN thickness was significantly increased in both somatosensory cortex and hippocampus of 22‐month‐old animals compared with 10‐month‐old animals (cortex: 10 m, 3.6 vs. 22 m, 5.1, *p* < 0.0001; hippocampus: 10 m, 2.6 vs. 22 m, 3.4, *p* < 0.0001). Notably, 4‐MU treatment restored PNN thickness to levels comparable to those observed in the younger group (cortex: 22 m + 4MU, 3.7, *p* < 0.0001; hippocampus: 22 m + 4MU, 3.8, *p* = 0.037) (Figure 1F,G).

To examine whether this reduction influenced memory performance, we evaluated SOR memory in the three groups of mice. Memory was tested at 6 h and 24 h delays between familiarization and testing, intervals that require consolidation, with 24 h representing a benchmark for robust long‐term memory in rodents (Villar et al. 2017; Cinalli Jr. et al. 2020). As expected, 22 m aged mice performed worse than 10 m adults at both intervals, though the differences did not reach significance (6 h: 10 m, 0.18 ± 0.12 vs. 22 m, 0.06 ± 0.15, *p* = 0.23; 24 h: 10 m, 0.13 ± 0.12 vs. 22 m, −0.05 ± 0.3, *p* = 0.33). 4‐MU‐treated aged mice showed improved performance at both delays. At 6 h, treated aged mice outperformed both 10 m and 22 m control groups (22 m + 4MU: 0.36 ± 0.11; *p* = 0.02 vs. 10 m; *p* < 0.0001 vs. 22 m) (Figure 1H). At 24 h, while not statistically significant, a clear trend toward improvement was observed (22 m + 4MU: 0.14 ± 0.22; *p* = 0.99 vs. 10 m; *p* = 0.08 vs. 22 m) (Figure 1I). Together, these findings indicate that oral 4‐MU treatment attenuates age‐associated PNN accumulation and alleviates aspects of age‐related cognitive decline.

### 3.2 Aging‐Induced Astrocytic Activation Is Suppressed by 4‐MU Treatment

We next investigated the effect of 4‐MU on chronic inflammation in aging. Given that neuroinflammaging has been reported to predominantly affect white matter regions (Raj et al. 2017; Hahn et al. 2023), we also included the corpus callosum in our assessment of neuroinflammatory changes in addition to the somatosensory cortex and hippocampus (Figure 2A). Our analysis revealed region‐specific patterns of astrocytic changes with aging (Figure 2B).

**FIGURE 2:**
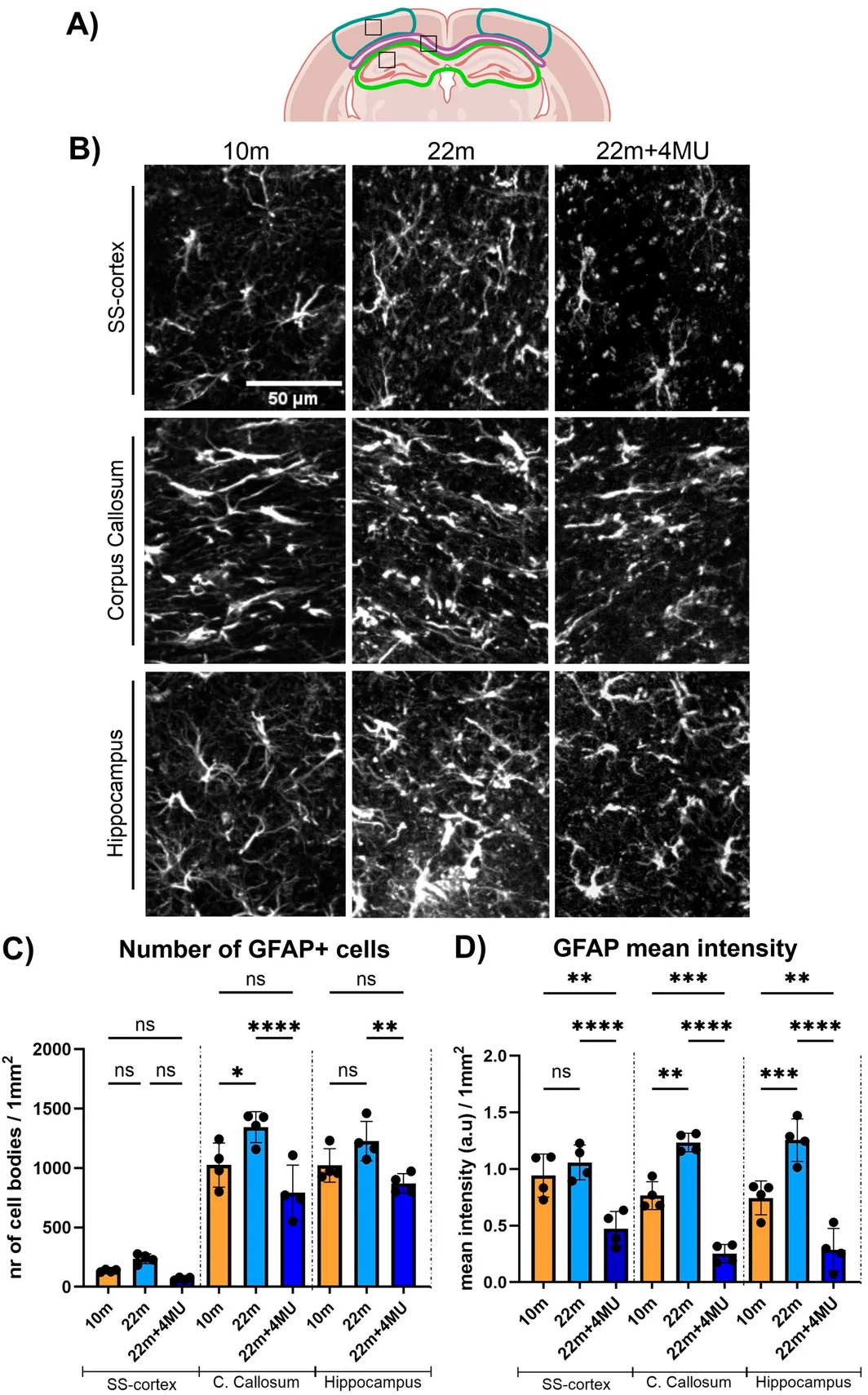
Astrocytes cell number and mean fluorescence intensity in cortex, corpus callosum, and hippocampus. (A) Schematic representation of the brain regions analyzed for neuroinflammatory changes. (B) Representative GFAP immunofluorescence images in the indicated areas. (C, D) Quantification of GFAP^+^ cell density and mean fluorescence intensity, respectively. Values are mean ± SD; ordinary one‐way ANOVA; *p < 0.05, **p < 0.01, ***p < 0.001, ****p < 0.0001.

In 22 m animals, GFAP‐positive cell density did not differ between the somatosensory cortex and hippocampus but was significantly increased in the corpus callosum compared with 10 m controls (10 m: 1025 ± 185 vs. 22 m: 1345 ± 130, *p* = 0.01). This increase was restored by 4‐MU treatment, with values comparable to 10 m controls (22 m + 4MU: 792 ± 232; *p* < 0.0001 vs. 22 m; *p* = 0.17 vs. 10 m) (Figure 2C). Similarly, GFAP intensity was significantly elevated in aged animals in the corpus callosum (10 m: 0.76 vs. 22 m: 1.23, *p* = 0.0014) and hippocampus (10 m: 0.74 vs. 22 m: 1.25, *p* = 0.0005), with a similar trend in the somatosensory cortex. Notably, 4‐MU treatment reduced GFAP intensity across all regions, reaching levels below those observed in both 22 and 10 m controls (Figure 2D).

Morphological analysis revealed reduced astrocytic arborization in aged animals in the somatosensory cortex and hippocampus across multiple process length intervals (Figure 3A–C), accompanied by pronounced soma hypertrophy in the corpus callosum (10 m: 14.0 ± 6.5 vs. 22 m: 44.7 ± 29.2, *p* < 0.0001) (Figure 3D,E). These age‐related alterations were largely normalized by 4‐MU treatment. In treated aged animals, arborization differed from 10 m controls only at isolated length intervals, whereas other intervals showed increased complexity compared with 22 m age‐matched controls. Likewise, soma area in the corpus callosum was restored to levels comparable to younger animals (22 m + 4MU: 19.5 ± 7.5; *p* = 0.42 vs. 10 m; *p* < 0.0001 vs. 22 m).

**FIGURE 3:**
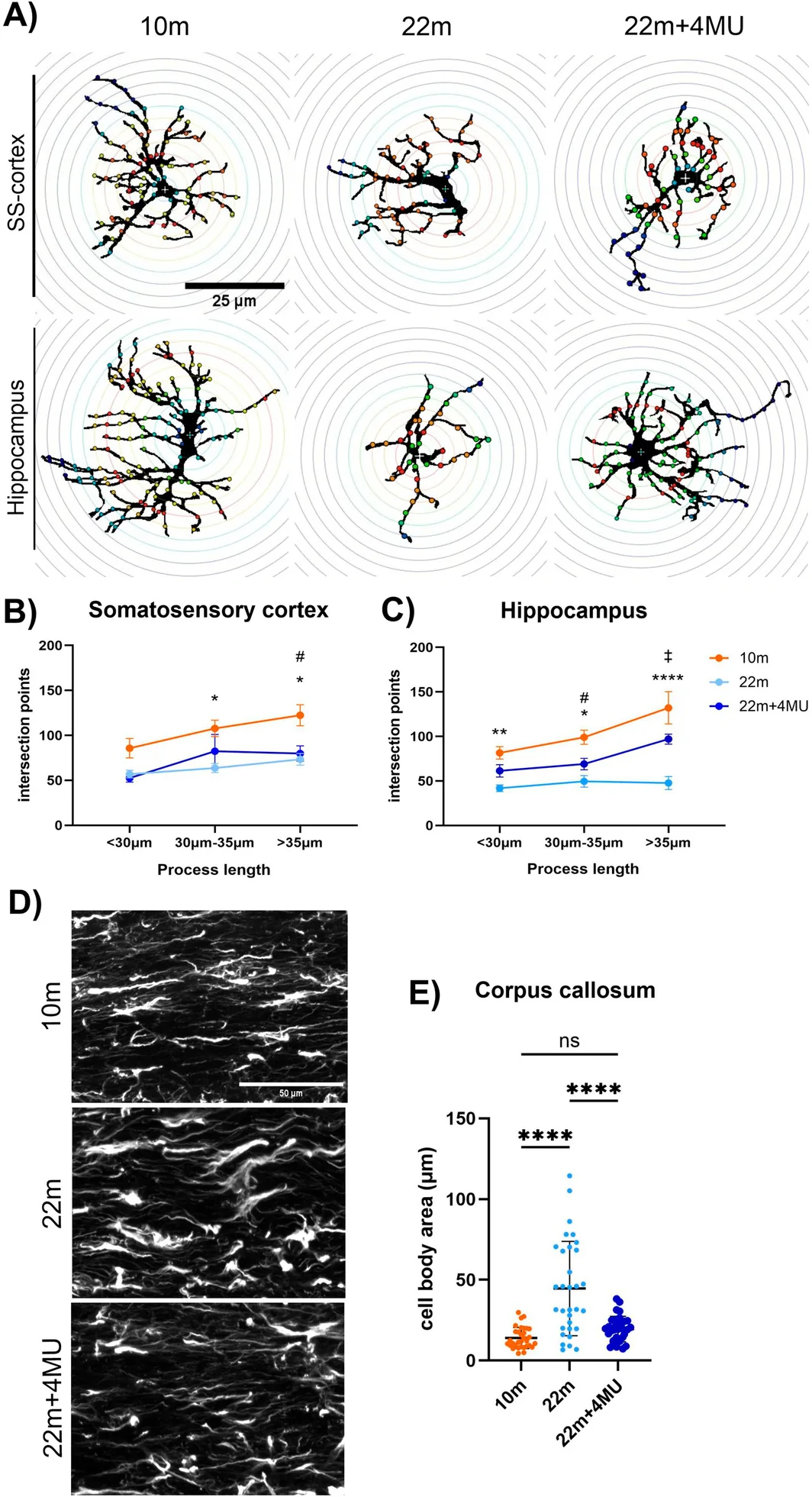
Morphological analysis of astrocytes. (A) Representative images of individual GFAP+ astrocytes assessed using the Scholl method in the somatosensory cortex and hippocampus. (B, C) Quantification of the number of intersection points per cell. (D) Representative images of astrocytes in the corpus callosum, and (E) quantification of soma area across the three animal groups. Data are presented as mean ± SD and were analyzed by one‐way ANOVA; *p < 0.05, **p < 0.01, ****p < 0.0001 (10 m vs. 22 m); ^#^ *p* < 0.05 (10 m vs. 22 m + 4MU); ^‡^ *p* < 0.05 (22 m vs. 22 m + 4MU).

Together, these findings indicate that aging induces region‐specific astrogliosis, with white matter regions being particularly vulnerable, showing an increased number of GFAP positive cells, GFAP intensity, and soma hypertrophy. Treatment with 4‐MU effectively counteracted these changes, indicating a pronounced astrocytic immunomodulatory effect and suggesting its potential to mitigate aging‐associated astrogliosis.

### 3.3 Aging‐Induced Microglia Activation Is Restored by 4‐MU Treatment

Another major contributor to neuroinflammaging is microglial reactivity (Von Bernhardi and Eugenín 2025). We next examined whether 4‐MU treatment could counteract aging‐associated microglial alterations. We therefore analyzed Iba1 positive cell density, Iba1 signal intensity (Figure 4A), and performed a morphological assessment of microglial arborization (Figure 5A) in the somatosensory cortex, hippocampus, and corpus callosum. In contrast to the astrocytic changes described above, aging was associated with a significant increase in both Iba1‐positive cell density and Iba1 intensity across all brain regions examined. Compared with 10 m, 22 m mice showed increased numbers of Iba1‐positive cells in the somatosensory cortex (10 m: 278 ± 15 vs. 22 m: 403 ± 44, *p* = 0.004), corpus callosum (10 m: 252 ± 31 vs. 22 m: 366 ± 62, *p* = 0.01), and hippocampus (10 m: 300 ± 20 vs. 22 m: 403 ± 38, *p* = 0.02) (Figure 4B). Similarly, Iba1 intensity was significantly elevated in aged animals in all regions (ss‐cortex: 10 m, 0.65 vs. 22 m, 1.34, *p* = 0.005; corpus callosum: 10 m, 0.49 vs. 22 m, 1.50, *p* < 0.0001; hippocampus: 10 m, 1.00 vs. 22 m, 2.96, *p* < 0.0001) (Figure 4C). Notably, 4‐MU treatment significantly reduced both Iba1‐positive cell density and Iba1 intensity, restoring values to levels comparable to 10 m adults (cell density: ss‐cortex, 289 ± 32, *p* = 0.01 vs. 22 m; corpus callosum, 172 ± 66, *p* < 0.0001 vs. 22 m; hippocampus, 284 ± 60, *p* = 0.007 vs. 22 m; intensity: ss‐cortex, 0.15, *p* < 0.0001 vs. 22 m; corpus callosum, 0.57, *p* = 0.0001 vs. 22 m; hippocampus, 0.46, *p* < 0.0001 vs. 22 m).

**FIGURE 4:**
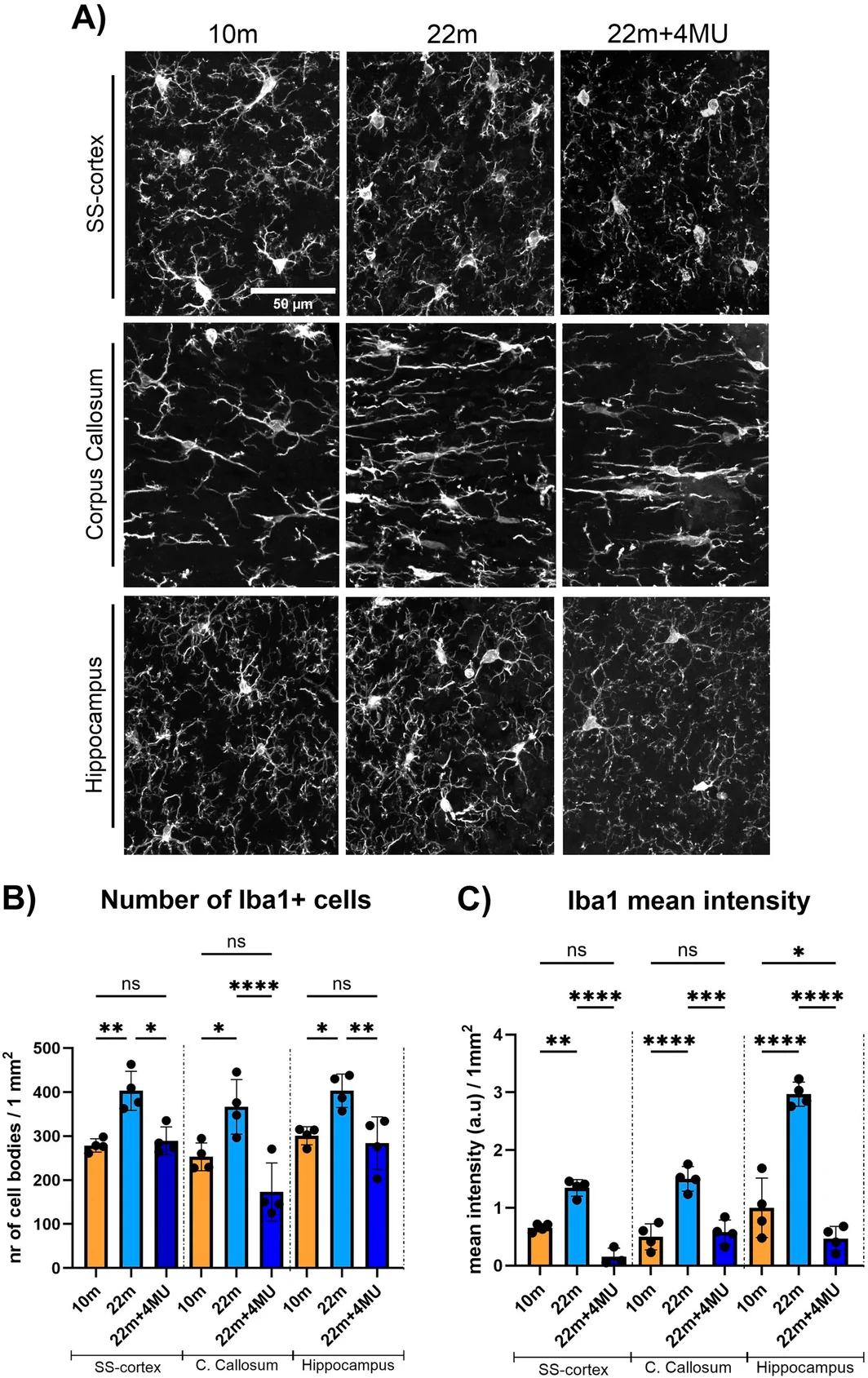
Microglia cell number and mean fluorescence intensity across brain regions. (A) Representative Iba1 staining in the somatosensory cortex, corpus callosum, and hippocampus. (B, C) Quantification of Iba1^+^ cell density and mean fluorescence intensity, respectively. Data are mean ± SD; ordinary one‐way ANOVA; *p < 0.05, **p < 0.01, ***p < 0.001, ****p < 0.0001.

**FIGURE 5:**
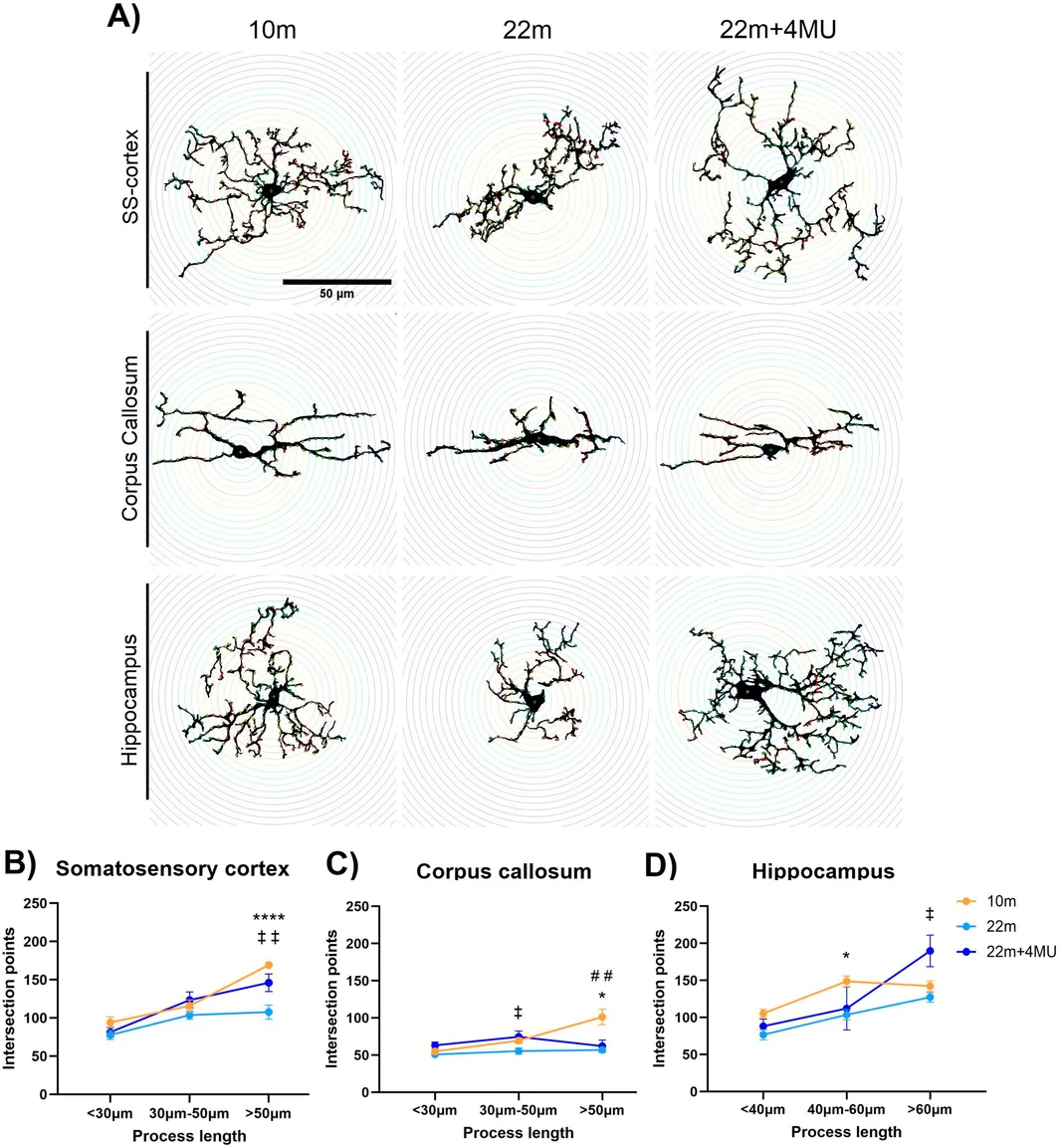
Morphological analysis of microglia. (A) Representative images of individual Iba1^+^ microglia used for morphological assessment. (B–D) Quantification of the number of intersection points per cell in the somatosensory‐cortex, corpus callosum, and hippocampus, respectively. Values are presented as mean ± SD; ordinary one‐way ANOVA; *p < 0.05, ****p < 0.0001 (10 m vs. 22 m); ^##^ *p* < 0.01 (10 m vs. 22 m + 4MU); ^‡^ *p* < 0.05, ^‡‡^ *p* < 0.01 (22 m vs. 22 m + 4MU).

Microglial morphology was also markedly altered with aging. Sholl analysis revealed reduced arborization in 22 m mice across all regions, consistent with a transition toward a reactive phenotype. In contrast, 4‐MU‐treated aged animals exhibited arborization patterns comparable to 10 m controls, with only a minor reduction observed at a single interval in the corpus callosum, and significantly greater complexity than age‐matched untreated animals in all regions (Figure 5B–D).

Together, these findings suggest that 4‐MU alleviates key age‐related microglial alterations across all examined regions, consistently reducing activation and restoring arborization, indicative of a broad modulatory influence on microglial remodeling with age.

### 3.4 Peripheral Immune Cell Infiltration Increases in Aged Brains and Is Normalized by 4‐MU

With aging, the BBB becomes more permeable, facilitating infiltration of peripheral immune cells. HA and CS have been implicated in immune cell recruitment, and 4‐MU has been reported to reduce immune cell infiltration across multiple organs (McKallip et al. 2013; Suarez‐Fueyo et al. 2019; Bosi et al. 2022; Galkina et al. 2022). In the CNS, this phenomenon is less well characterized. Existing evidence suggests that 4‐MU can also limit infiltration, but only in disease or injury models (Kuipers, Rieck, et al. 2016; Štepánková et al. 2023; Mueller et al. 2014), not under normal aging conditions where BBB alterations follow different dynamics. To address this gap, we examined peripheral immune cell infiltration into the brains of 10 m adult, aged, and aged 4‐MU‐treated animals. We quantified infiltrating immune cells using CD45 as a pan‐leukocyte marker. Because CD45 is also expressed by microglia, we combined CD45 immunolabeling with Iba1 to identify peripheral immune cells as CD45‐positive/Iba1‐negative. Analyses were performed in the somatosensory cortex, corpus callosum, and hippocampus (Figure 6A). Results showed a significant increase in CD45‐positive/Iba1‐negative cells in 22 m aged mice compared with 10 m controls in the corpus callosum (10 m: 8 ± 4, 22 m: 17 ± 5, *p* = 0.001) while the somatosensory cortex and hippocampus showed no substantial changes. These findings highlight that age‐related immune infiltration is most prominent in white matter regions, consistent with other hallmarks of neuroinflammaging. Importantly, 4‐MU treatment markedly reduced the elevated immune cell numbers in the corpus callosum, restoring them to levels observed in 10 m animals (5 ± 3, *p* = 0.8 vs. 10 m, *p* < 0.0001 vs. 22 m) (Figure 6B). Our results indicate that 4‐MU can reduce age‐related peripheral immune cell infiltration into the corpus callosum, which may limit inflammatory signaling, thereby creating a more permissive environment for neuronal health and neuroplasticity.

**FIGURE 6:**
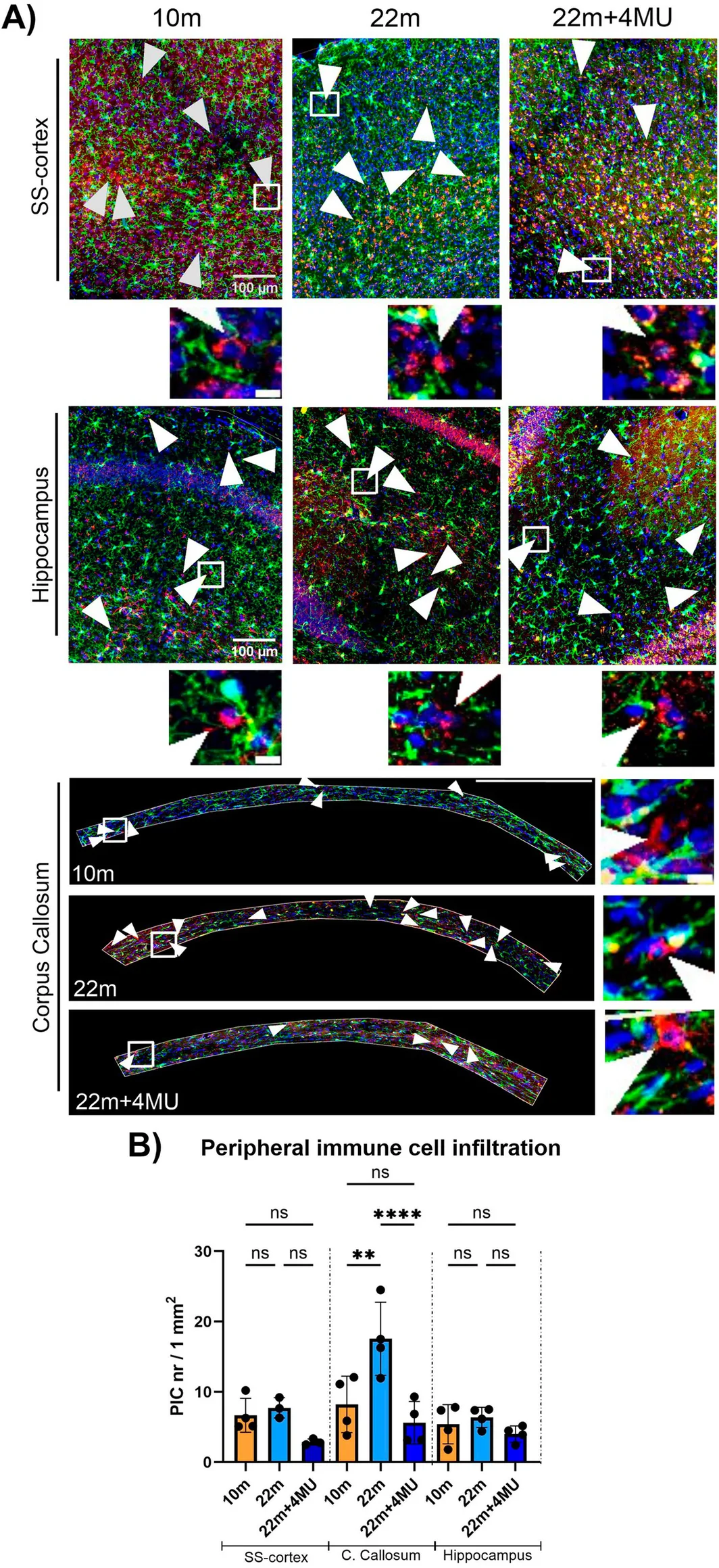
Peripheral immune cell (PIC) infiltration in cortex, corpus callosum, and hippocampus. (A) Representative images of CD45 (red), Iba1 (green), and DAPI (blue) staining in the indicated regions. Arrows mark CD45^+^Iba1^−^ cells and the insets show a magnified view of one such cell. (B) Quantification of CD45^+^Iba1^−^ cells per mm^2^ in the indicated areas. Data are presented as mean ± SD and were analyzed using ordinary one‐way ANOVA; **p < 0.01, ****p < 0.0001.

### 3.5 Long‐Term Oral 4‐MU Treatment Is Well Tolerated in Aged Mice

Numerous studies have demonstrated that 4‐MU is a well‐tolerated compound with minimal adverse effects (Dubisova et al. 2022; Štěpánková et al. 2023; Nagy et al. 2024). However, given that our study involved old mice, whose physiology may differ from animals in previous studies, we evaluated multiple parameters to assess 4‐MU safety in aged animals (Figure 7). The body weight of the animals showed a significant reduction in 4‐MU–treated mice compared to controls, similar to previous studies (Figure 7A). As HA is a key component for cartilage functions, motor and joint mobility were assessed by grip strength (Figure 7B) and rotarod tests (Figure 7C) and found no differences between treated and 22 m control mice. Hematological analyses, including total leukocytes, erythrocytes, platelets, reticulocytes, and leukocyte subpopulations (neutrophils, lymphocytes, monocytes, eosinophils, and basophils), showed values within the expected physiological range, with no differences between treated and control mice (Figure 7D). Finally, we assessed bile acids and glucose levels in blood and urine, parameters previously reported to be influenced by 4‐MU treatment. Although no significant changes were detected, a trend toward increased levels exceeding the physiological range (Unno et al. 2023; Hönes 2024; Norman et al. 2024; Zheng et al. 2024) was observed, particularly in glycosuria (Figure 7E–H). Taken together, these results indicate that long‐term oral administration of 4‐MU is generally safe in old mice, causing no major hematological, biochemical, or functional alterations within the parameters assessed in this study.

**FIGURE 7:**
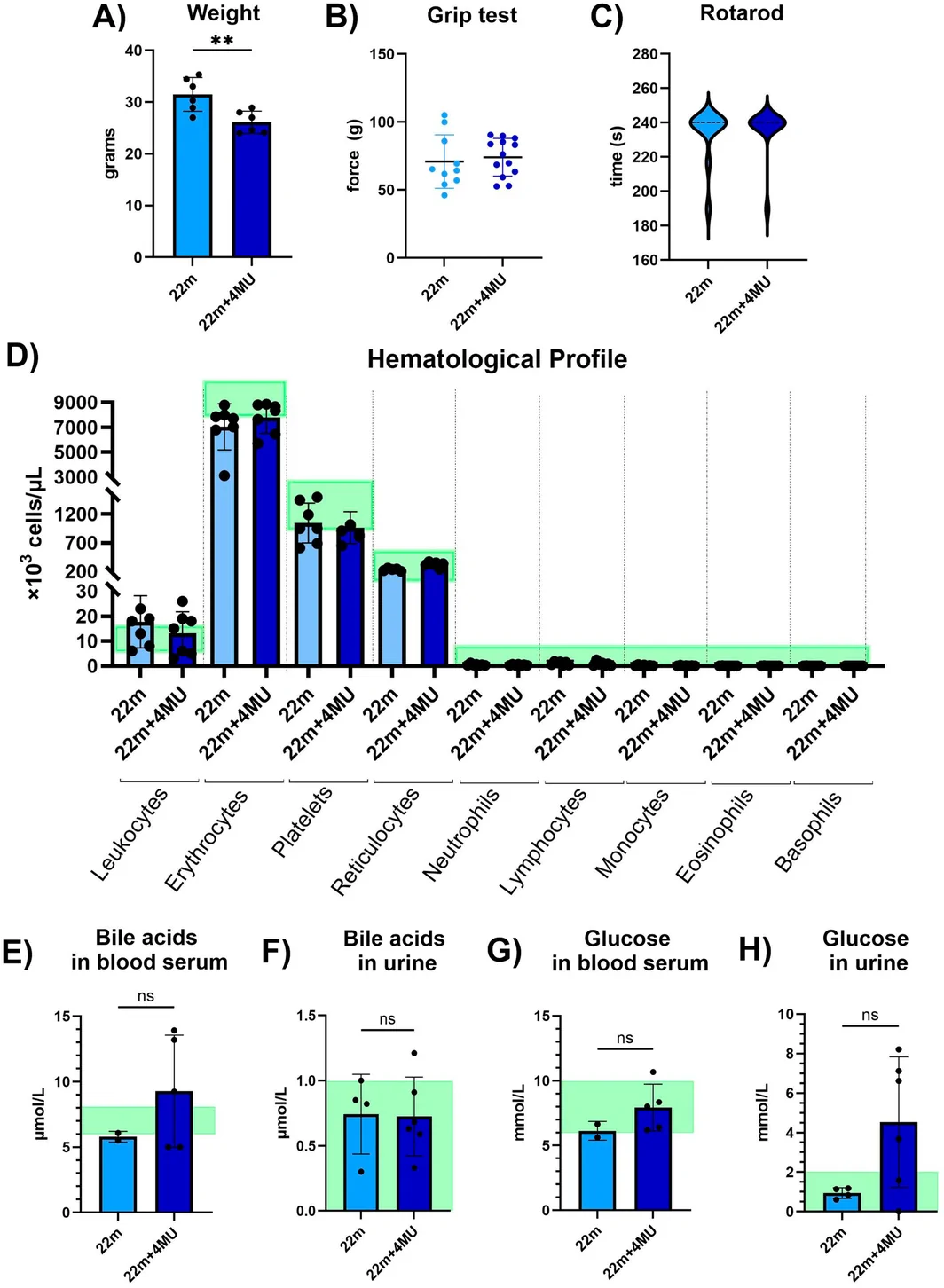
Assessment of safety parameters in aged mice following long‐term 4‐MU treatment. (A) Body weight measured at the end of the experimental period. (B) Forelimb grip strength. (C) Rotarod performance. (D) Hematological profile. (E–H) Bile acids and glucose concentrations in blood and urine, respectively. Physiological range is indicated in green. Data are presented as mean ± SD. Statistical comparisons between old control and old 4‐MU–treated groups were performed using unpaired t‐test with significance indicated as **p < 0.01.

## 4 Discussion

Here, we show that aging is associated with significant changes in PNN properties in the brain regions examined, including increased intensity and thickness. In addition, it also led to region‐specific neuroinflammatory changes in astrocytes, microglia, and infiltrating peripheral immune cells. Chronic 6 months 4‐MU treatment attenuated these alterations and improved object recognition memory, suggesting complementary effects of PNN modulation and reduced neuroinflammation. These results position the HA and CS as a central integrator of plasticity and neuroinflammation in the aging brain.

The ECM plays key regulatory roles in neuronal and glial function through diverse and dynamic interactions. HA, a major glycosaminoglycan distributed across gray and white matter, signals through receptors such as CD44, RHAMM (HMMR), LYVE‐1, ICAM‐1, and TLR2/4 to influence proliferation, differentiation, migration, activation states, and process extension in stem cells, neurons, astrocytes, and microglia (Peters and Sherman 2020). HA also forms the structural backbone of PNNs, specialized ECM assemblies that support circuit stability and excitatory–inhibitory balance. Furthermore, HA contributes to endothelial barrier regulation and lymphocyte extravasation (Galkina et al. 2022). In parallel, CSPGs, another major class of ECM components, undergo progressive remodeling during aging that is associated with reduced structural plasticity and altered network stability (Fawcett 2015). Through their sulfated glycosaminoglycan chains, CSPGs interact with receptors such as PTP*σ*, LAR, and NgR1 complexes to constrain synaptic remodeling and neurite dynamics, thereby contributing to the maintenance of stable but less adaptable circuits (Fisher et al. 2011; Dickendesher et al. 2012). Beyond their established effects on neuronal plasticity, CSPGs also regulate neuroimmune responses by modulating microglial and astrocytic states (Hirano et al. 2025; Dyck and Karimi‐Abdolrezaee 2015; Siebert et al. 2014) as well as peripheral immune cell infiltration (Pu et al. 2020; Stephenson and Yong 2018; Francos‐Quijorna et al. 2022). Together, HA and CSPGs form complementary regulatory axes within the compacted‐PNNs and diffuse ECM that influence both neural plasticity and neuroimmune homeostasis across the lifespan.

Previous studies indicate that age‐related PNN remodeling is highly region‐ and component‐dependent (Colon et al. 2025; Ueno et al. 2018, 2019), with increases in WFA‐positive PNN density reported in sensory cortices and hippocampal CA2 (Karetko‐Sysa et al. 2014; Mafi et al. 2020; Lehner et al. 2024), whereas other cortical and striatal regions show little or no change, or even reductions in PNN‐related measures (Barahona et al. 2025). Moreover, aging alters the molecular composition of PNNs, including changes in CS sulfation and aggrecan/HA organization (Foscarin et al. 2017; Yang et al. 2021). In line with the region‐ and parameter‐dependent nature of age‐related PNN remodeling, our results likewise revealed a heterogeneous pattern, with some regions and PNN parameters showing no significant age‐related changes, whereas others exhibited clear trends or significant alterations. Notably, chronic 4‐MU treatment consistently shifted the affected PNN parameters toward levels comparable to those observed in 10‐month‐old controls, suggesting that 4‐MU may attenuate age‐associated PNN remodeling. PNNs are central regulators of neuroplasticity and have been implicated in the closure of critical periods, ocular dominance plasticity, auditory relearning, fear extinction, and multiple forms of memory (Reichelt et al. 2019). By depleting HA and CS, 4‐MU is thought to weaken the structural integrity and inhibitory signaling capacity of PNNs, thereby shifting the ECM toward a more permissive state for structural plasticity. In our previous work, we showed that oral 4‐MU reduces PNN intensity and enhances object recognition memory in young adult mice (Dubisova et al. 2022). Experimental PNN degradation using chondroitinase‐ABC, which digests CSPGs, substantially prolongs long‐term object recognition memory in both wild‐type and MAPT mouse models of dementia (Romberg et al. 2013; Yang et al. 2015). Similarly, mice lacking Crtl1/Hapln1 in the CNS display markedly extended recognition memory compared with controls (Romberg et al. 2013). Age‐related cognitive decline primarily affects the acquisition of new long‐term memories, while retrieval of remote memories remains largely preserved (Brito et al. 2023). Because the SOR task specifically evaluates acquisition of new information, it is well suited for aging studies. Notably, aged mice treated with 4‐MU showed enhanced object recognition, surpassing 10 m controls at the 6‐h interval, and approaching 10 m performance at 24 h. These results are consistent with previous literature and suggest that modulating HA and CS metabolism may represent a promising strategy to mitigate age‐related cognitive impairment.

Regarding neuroinflammaging, transcriptomic analyses of the aged human brain have shown that glial cells, rather than neurons, undergo the majority of age‐associated transcriptional changes (Soreq et al. 2017). Astrocytes participate in the tetrapartite synapse, support neurons, interact with vasculature, and contribute to BBB integrity. Microglia serve as the innate immune cells of the CNS and, beyond their injury response, have essential homeostatic roles including regulation of synapse formation, maturation, and elimination, as well as modulation of neurogenesis, myelination, and neuroplasticity. With aging, both cell types show reduced homeostatic capacity and elevated inflammatory reactivity, increasing neuronal vulnerability and predisposing the brain to age‐related decline. Region‐specific glial changes have been described, typically characterized by stable or increased cell numbers, elevated marker expression, hypertrophic somata, and reduced ramification in both astrocytes (Clarke et al. 2018; Cotto et al. 2019) and microglia (Raj et al. 2017; Colon et al. 2025; Ana 2024), though with distinct patterns across brain regions (Salas et al. 2020). In line with the literature, we observed the following aging‐related alterations:

- *In the somatosensory cortex*: It shows minimal neuroinflammatory changes. Astrocyte numbers and GFAP expression remained stable, although arborization was reduced. Microglia displayed increased numbers, elevated Iba1 expression, and reduced branching.
- *In the hippocampus*: It shows moderate neuroinflammatory changes. Astrocyte numbers were unchanged, but GFAP expression increased and arborization decreased. Microglia exhibited elevated numbers and marker expression with reduced arborization.
- *In the corpus callosum*: It shows the most pronounced alterations. Astrocyte number and GFAP expression were significantly increased, with hypertrophic morphologies consistent with reactivity. Microglia showed robust increases in number and marker intensity and substantial reductions in arborization.

These results mirror previous observations that white matter regions are, particularly, vulnerable to neuroinflammaging (Raj et al. 2017; Hahn et al. 2023), that the hippocampus exhibits more pronounced changes than neocortical areas such as the somatosensory cortex (Salas et al. 2020), and that microglia display more marked aging‐associated alterations than astrocytes, including in nonhuman primates (Salas et al. 2020). Chronic 4‐MU treatment in aged mice effectively attenuated these neuroinflammatory signatures across all examined regions, restoring astrocytic and microglial parameters to levels comparable to 10 m controls. Several in vitro (Chistyakov et al. 2020) and in vivo (Kuipers, Rieck, et al. 2016; Štepánková et al. 2023; Syková et al. 2025) studies have shown that 4‐MU attenuates glia inflammatory responses, supporting our observations.

Aging is also associated with BBB deterioration and increased permeability. Tissue‐resident T cells have been described to accumulate in the white matter of older individuals (Jurcau et al. 2024). In our study, the corpus callosum was the only region displaying higher infiltration of peripheral immune cells with aging. HA contributes to immune cell recruitment by binding CD44 on vascular endothelium facilitating leukocyte adhesion, and HA fragments signal through TLR2/4 to promote inflammatory gene expression (Al‐Ahmad et al. 2019). On the other hand, CSPGs have the ability to bind and present chemokines/cytokines within the ECM, creating localized inflammatory gradients, interacting with adhesion receptors such as selectins, CD44, and integrins, influencing leukocyte migration, and activating innate immune signaling pathways, including TLR4, thereby influencing immune cell recruitment and activation (Pu et al. 2020; Stephenson and Yong 2018; Francos‐Quijorna et al. 2022). 4‐MU has been shown to reduce immune cell infiltration in multiple organs (McKallip et al. 2013; Suarez‐Fueyo et al. 2019; Bosi et al. 2022) and in the CNS during EAE (Mueller et al. 2014), consistent with our finding that 4‐MU reduces peripheral immune cells infiltration in the aging brain.

Together, these results suggest that 4‐MU exerts broad immunomodulatory effects through modulation of HA and CS in the PNN‐related regulation pathways, mitigating age‐related neuroinflammation and potentially acting in concert with reduced PNN density to support cognitive improvement.

4‐MU may exert direct cellular effects independent of ECM remodeling, and future studies will be required to disentangle ECM‐mediated versus cell‐intrinsic mechanisms. In addition, 4‐MU may influence other cell types, such as stem or progenitor cells, which are relevant for memory‐related processes, and infiltrating macrophages, a key cellular component of inflammaging, as well as additional brain regions that were not examined in this study. Moreover, recent work shows that 4‐MU, by modulating ECM components, alters PNNs and astrocytes (consistent with our findings) and induces changes in brain microstructure, increasing extracellular space fraction and modifying diffusion parameters reminiscent of early developmental stages associated with higher plasticity (Syková et al. 2025). These observations point to additional mechanisms of 4‐MU action that remain unexplored in our study, underscoring the need for further investigation into its broader effects.

Regarding safety, previous studies reported no major adverse effects of 4‐MU and reversibility of observed changes after washout (Štěpánková et al. 2023; Nagy et al. 2024; Kuipers, Nagy, et al. 2016). In this study, treated animals exhibited significant weight loss, consistent with prior reports linking 4‐MU to altered glucose and triglyceride metabolism and enhanced brown adipose tissue thermogenesis (Nagy et al. 2024; Grandoch et al. 2019; Tsitrina et al. 2023). Importantly, rotarod and grip strength performance remained unaffected, and hematological parameters were stable. We noted a mild, nonsignificant increase in circulating bile acids, aligning with the known pharmacodynamic action of 4‐MU on bile acid secretion. Glycosuria occurred without hyperglycemia, suggesting a tubular alteration of glucose reabsorption rather than a systemic effect. Similar findings have been reported elsewhere, with no kidney histopathology and normalization of biochemical markers posttreatment (Štěpánková et al. 2023).

In conclusion, PNN accumulation and neuroinflammation are well‐established features of CNS aging that contribute to cognitive decline. Our findings suggest that CS and HA function as a multifacet regulator in the aging brain, orchestrating structural, cellular, and vascular‐immune dynamics that together influence neuronal health and plasticity. 4‐MU, an inhibitor of HA and CS synthesis with an established pharmacokinetic profile in animals and humans, is clinically approved for biliary cholestasis and under investigation for immune and oncological disorders, making it an attractive candidate for repurposing (Fedorova et al. 2025). Our work characterizes PNN and neuroinflammation during normal aging and shows that chronic oral 4‐MU administration mitigates these changes and increases object recognition memory without evidence of severe adverse effects. These findings support the potential of 4‐MU to ameliorate age‐related cognitive decline through coordinated functions linked to ECM modulation.

## Author Contributions

**Anda Cimpean:** conceptualization, data curation, formal analysis, investigation, visualization, writing – original draft. **Jana Mizerakova:** conceptualization, investigation, data curation. **Noelia Martinez‐Varea**, **Dana Marekova**, **Lucia Machova Urdzikova:** investigation. **James W. Fawcett:** conceptualization, funding acquisition, methodology, writing – review and editing. **Pavla Jendelova:** conceptualization, funding acquisition, project administration, supervision, writing – review and editing. **Jessica C. F. Kwok:** conceptualization, funding acquisition, methodology, supervision, validation, writing – review and editing.

## Funding

This work was supported by Czech Science Foundation (GACR) 23‐05540S and Co‐financed from EU funds, project EXREGMED: CZ02.01.01/00/22‐008/0004562.

## Conflicts of Interest

The authors declare no conflicts of interest.

## Supporting information


**Figure S1:** Pixel intensity quartile analysis of WFA staining. (A) Fraction of WFA‐positive pixels assigned to each intensity quartile (Q1–Q4) in the somatosensory cortex and hippocampus across experimental groups. (B) Statistical analysis of the highest intensity quartile (Q4; top 25% brightest pixels) in the ss‐cortex and hippocampus. Data are presented as mean ± SD. *p < 0.05, **p < 0.01.

## Data Availability

The datasets presented in this study can be found in online repositories. The names of the repository/repositories and accession number(s) can be found below: https://zenodo.org/records/18223474?preview=1&token=eyJhbGciOiJIUzUxMiJ9.eyJpZCI6ImE3NTlkMThlLTY0NzEtNDI5Ni1iNTJmLTQ0MWY3YTk1NmY2MyIsImRhdGEiOnt9LCJyYW5kb20iOiI5YjJkZTQ1YzE2OTkwNzY1MGJhNWY4NzRlYTg3OTEzMyJ9.UNaU1YShaRJxj0uyt5aheJ_2Mjt1tjKLwIV0_6Fmf2zSY‐ChInADkM1hmcdDQIjjlJyFgJJAOm7G‐70c09kvLA.
