# Supplementary material for: 4‐Methylumbelliferone Restores Age‐Related Changes in Perineuronal Nets, Memory, and Neuroinflammation
Source: Glia. 2026 Sep 30;74(12):e70233. doi: 10.1002/glia.70233 (PMC13625197; doi:10.1002/glia.70233)
Supplement: Supplementary file 1 [file GLIA-74-0-s001.pdf]

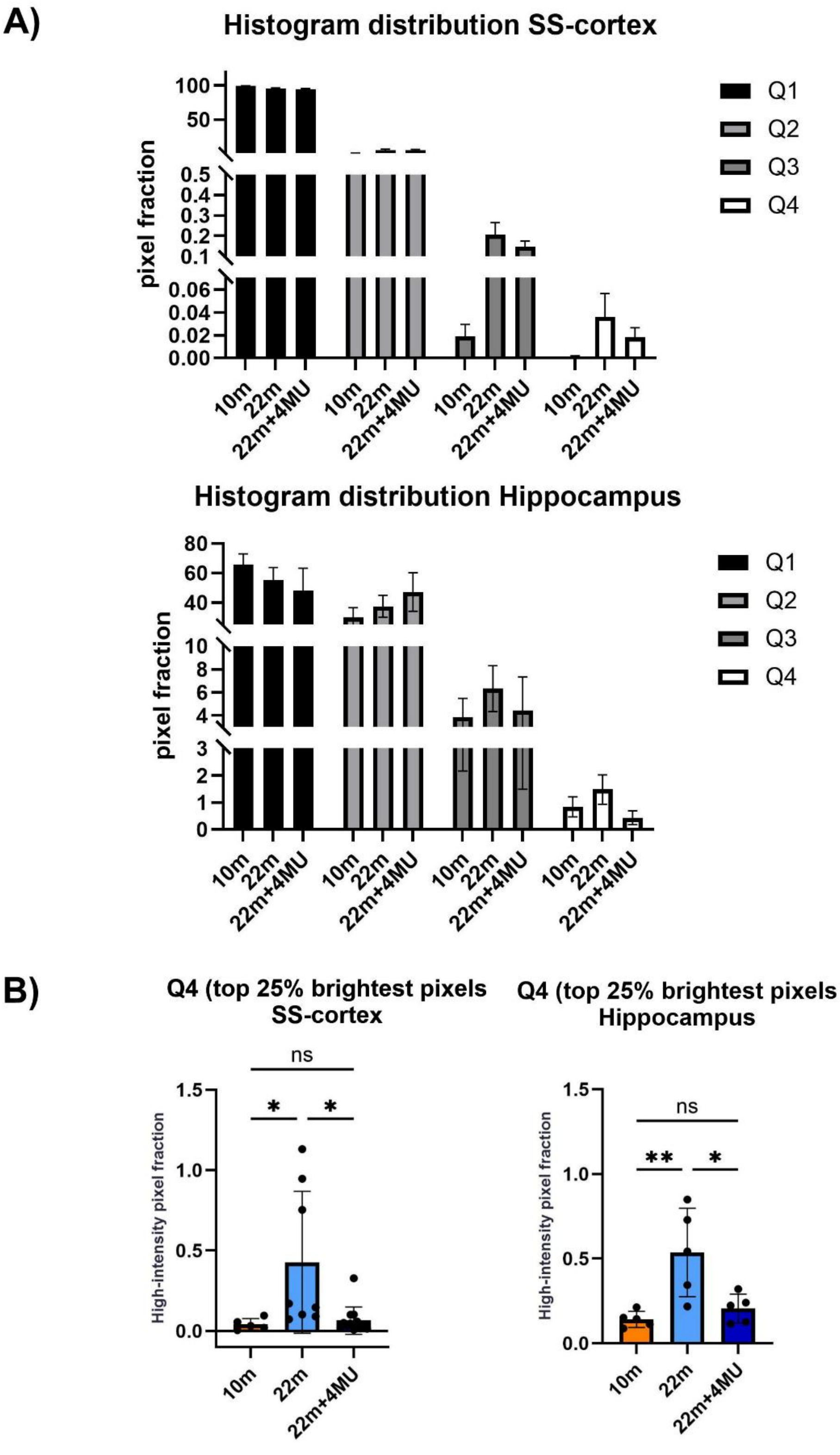

Supplementary Figure 1. Pixel intensity quartile analysis of WFA staining. (A) Fraction of WFA-positive pixels assigned to each intensity quartile (Q1–Q4) in the somatosensory cortex and hippocampus across experimental groups. (B) Statistical analysis of the highest intensity quartile (Q4; top 25% brightest pixels) in the ss-cortex and hippocampus. Data are presented as mean ± SD. *p < 0.05, **p < 0.01.
